# Antidiabetic Flavonoids from Fruits of *Morus alba* Promoting Insulin-Stimulated Glucose Uptake via Akt and AMP-Activated Protein Kinase Activation in 3T3-L1 Adipocytes

**DOI:** 10.3390/pharmaceutics13040526

**Published:** 2021-04-09

**Authors:** Sung Ho Lim, Jae Sik Yu, Ho Seon Lee, Chang-Ik Choi, Ki Hyun Kim

**Affiliations:** 1 BK21 FOUR Team and Integrated Research Institute for Drug Development, College of Pharmacy, Dongguk University-Seoul, Goyang 10326, Korea; 93sho617@naver.com; 2 School of Pharmacy, Sungkyunkwan University, Suwon 16419, Korea; jsyu@bu.edu; 3 Integrated Research Institute for Drug Development, College of Pharmacy, Dongguk University-Seoul, Goyang 10326, Korea; ghtjsrhtn@naver.com

**Keywords:** type 2 diabetes, *Morus alba*, rutin, quercetin-3-*O*-β-d-glucoside, glucose uptake, lipid accumulation, 3T3-L1 adipocyte

## Abstract

*Morus alba* (Moraceae), known as white mulberry, has been used to treat fever, protect against liver damage, improve eyesight, and lower blood sugar levels in traditional oriental medicine. Few studies have been conducted on the antidiabetic compounds identified from *M. alba* and their underlying mechanisms of action. Consequently, in this study, the fruits of *M. alba* were investigated for potential antidiabetic natural products using 3T3-L1 adipocytes. Phytochemical analysis of the ethanolic extract of *M. alba* fruits, followed by high-performance liquid chromatography (HPLC), purification led to the isolation of two main compounds: rutin and quercetin-3-*O*-β-d-glucoside (Q3G). Long-term use of available drugs for treating type 2 diabetes ((T2D) is often accompanied by undesirable side effects, which have generated increased interest in the development of more effective and safer antidiabetic agents. Examination of the isolated compounds, rutin and Q3G, for antidiabetic or anti-obesity properties or both in 3T3-L1 adipocytes demonstrated that they both improved glucose uptake via Akt-mediated insulin signaling pathway or AMP-activated protein kinase (AMPK) activation in 3T3-L1 adipocytes. The compounds also showed a positive effect on lipid accumulation in adipocytes, suggesting that glucose uptake occurred through activation of the Akt and AMPK signaling pathway without inducing adipogenesis. Taken together, our findings suggest that rutin and Q3G in *M. alba* fruits have the potential to induce fewer side effects such as weight gain, and these active compounds could be potential therapeutic candidates for the management of T2D.

## 1. Introduction

Type 2 diabetes (T2D) is a metabolic disorder that affected an estimated 2.8% of the world population in 2000, and this incidence is expected to rise to 4.4% by 2030 [1]. T2D affects metabolic organs, such as the muscle, liver, and adipose tissue, and arises from a defect in insulin usage. Hyperglycemia is a clinical marker of T2D, along with body and organ weight gain and insulin resistance. In particular, insulin resistance, which is a specific feature of T2D, often causes elevated blood glucose accompanied by increased blood pressure and lipid levels. This can lead to the development or exacerbation of chronic diseases such as obesity, hypertension, atherosclerosis, liver failure, and cancer. In this regard, enhancing glucose uptake to organs and tissues in the body is one of the important strategies for maintaining appropriate blood glucose levels in T2D patients.

The maintenance of glucose homeostasis is essential for glucose uptake in insulin-responsive tissues such as skeletal muscles and adipose tissues in the human body. It is mediated by some members of a family of glucose transporters (GLUTs) [2], which have different substrate affinities, tissue-specific expression, and regulatory processes. GLUT1 and GLUT4 are two major glucose transporter isoforms expressed in adipose tissue, and many investigations have shown that they play a pivotal role in peripheral glucose regulation [2]. GLUT1 is a constitutive glucose transporter mainly located in plasma membranes and intracellular membranes, whereas the expression of GLUT4 is dependent on the amount of insulin and it is sequestered in intracellular vesicles under insulin insufficiency [3,4]. The phosphatidylinositol 3′ kinase (PI3K)/Akt and AMP-activated protein kinase (AMPK) pathways in adipocytes trigger GLUT4 translocation to the plasma membrane by insulin [3,5]. In several studies, overexpression of GLUT1, GLUT4, or both was found to cause hypoglycemia, whereas downregulation of GLUT4 led to insulin resistance [4]. It is known that increased expression and plasma membrane translocation of GLUT1 and GLUT4 improve glucose transport and utilization, thereby lowering blood glucose levels [6]. Glucose transferred to cells is metabolized or converted into its storage form, glycogen. Therefore, simultaneously enhancing glucose uptake and glycogen synthesis is considered the most beneficial therapeutic approach to overcome insulin resistance in T2D patients [7,8,9]. After binding to its cognate receptor, insulin activates the intrinsic protein tyrosine kinase activity and induces phosphorylation of tyrosine residues [10]. These activated receptors recruit and phosphorylate substrate molecules. Insulin receptor substrate (IRS) 1 and IRS2 are adapter molecules that play a central role in the coupling of PI3K/Akt and mitogen-activated protein kinase (MAPK) downstream kinases [11]. Tyrosine-phosphorylated IRS1/2 recruits the heterodimeric p85/p110 PI3K at the plasma membrane, where it produces the lipid second messenger phosphatidylinositol (3,4,5)-trisphosphate (PIP3), which in turn activates the serine/threonine phosphorylation cascade of PH domain-containing proteins [12]. PIP3 targets include phosphoinositide-dependent protein kinase 1 (PDK1), serine/threonine protein kinase B (PKB), also known as Akt, and atypical protein kinase C ζ and λ isoforms [13,14]. Akt plays an important role in multiple cellular processes associated with cell growth, survival, proliferation, and metabolism [15]. Akt also plays a key role in insulin-stimulated glucose uptake in the muscle and adipose tissue. In contrast, it inhibits glucose release in hepatocytes [16]. Glucose uptake in peripheral tissues via Akt/PKB is mediated by the translocation of GLUTs to the cell membrane by the effect of insulin, which facilitates glucose uptake.

AMPK is a nutrient-sensing serine/threonine kinase that is activated when the cellular energy level is low (i.e., high intracellular AMP:ATP ratio) and regulates energy homeostasis in the body. Activated AMPK subsequently induces downstream signals and restores energy to normal levels by stimulating ATP generation, such as fatty acid oxidation, and inhibiting ATP-consuming processes (e.g., triglycerides and protein synthesis) [17]. Thus, advantageous effects on metabolic disorders, such as T2D and obesity, play a central role in AMPK activation. Some antidiabetic drugs such as metformin and thiazolidinediones (TZDs) activate the AMPK signaling pathway [18]. In addition, AMPK activation enhances GLUT4 translocation into the plasma membrane of 3T3-L1 adipocytes [19].

Over the past few decades, the number of obese patients has increased rapidly, making it one of the most serious public health problems worldwide [20]. Obesity is closely related to insulin resistance in T2D patients, since both disorders involve dysfunction of endocrine, inflammatory, neuronal, and cellular intrinsic pathways [21]. Therefore, obesity-associated insulin resistance is considered a major risk factor for T2D [22]. The well-known link between obesity and insulin resistance in T2D is adipocyte dysregulation [23]. Hyperplasia and hypertrophy of adipose tissues are regulated by the capacity for lipid storage in adipocytes [24]. When the storage capacity reaches a threshold level in adipose tissues, excess fat is redistributed to other organs such as the liver, pancreas, or muscles, causing insulin resistance and toxic reactions. Abnormal fat accumulation in adipose tissue alters the expression of several inflammatory factors or adipokines, some of which also affect the insulin sensitivity in other metabolic organs [25].

Hypoglycemic agents with various mechanisms, including TZDs, sulfonylureas, and biguanides, are currently on the market to control blood glucose levels and prevent long-term complications in T2D patients. However, due to the potential for drug-induced side effects, such as hypoglycemic episodes, gastrointestinal disorders, weight gain, lactic acidosis, and low-density lipoprotein (LDL) cholesterol elevation, there is an increasing demand for the discovery of novel, naturally derived substances that are safe and pharmacologically effective [26].

As part of an ongoing research project to discover bioactive natural products [27,28,29,30,31], we investigated candidate phytochemicals from the extract of fruits of *Morus alba* L. to explore the antidiabetic or anti-obesity potential by using 3T3-L1 adipocytes. *M. alba*, also known as white mulberry or “sang shu,” is a perennial plant belonging to the family Moraceae. This plant is native to China and is also widely cultivated in China, Japan, and Korea as an important food source for silkworms [32,33]. This plant grows numerous fruits with a sweet flavor, which are popularly consumed in various forms, including tea, beverages, and desserts, worldwide. Traditionally, the leaves, fruits, and bark of this plant have long been used to treat fever, protect against liver damage, improve eyesight, strengthen joints, facilitate the discharge of urine, and lower blood pressure [34]. Interestingly, in Korea and Japan, patients with diabetes consume mulberry leaves as an anti-hyperglycemic supplement [35], and the leaves are known to be effective against high blood pressure and hangover from alcohol and in lowering blood sugar levels related to diabetes [36]. Previous biological studies of *M. alba* have reported that its extracts exhibit diverse therapeutic properties, including antioxidant, antimicrobial, skin-whitening, cytotoxicity, anti-inflammatory, anti-hyperlipidemic, hepatoprotective, antidiabetic, and anti-obesity effects [37,38]. In addition, previous phytochemical studies on *M. alba* have demonstrated the presence of diverse chemical constituents including terpenoids, alkaloids, flavonoids (including chalcones and anthocyanins), phenolic acids, stilbenoids, and coumarins [38]. However, few previous studies have investigated the chemical constituents of *M. alba* with antidiabetic or anti-obesity effects and their underlying mechanisms of action, despite many studies on the pharmacological effects of *M. alba* extracts.

In this context, our group has focused on the potentially bioactive natural components of *M. alba*. In our previous chemical investigation of *M. alba* fruits, we identified a new oxolane derivative, odisolane, and five heterocyclic compounds [39], some of which showed significant antiangiogenic activity mediated by decreasing p-Akt, vascular endothelial growth factor (VEGF), and phosphorylated, extracellular-signal-regulated kinase (phospho-ERK) protein expression. Furthermore, we identified two indole acetic acid derivatives and flavonoids from *M. alba* fruits and found that a two-indole acetic acid derivative exhibited cytotoxicity against human cervical cancer HeLa cells through the activation of caspase-8 and -9, followed by cleavage of poly-ADP ribose polymerase (PARP) [40].

We also identified butyl pyroglutamate from *M. alba* fruits as a nephroprotective agent against cisplatin-induced kidney cell death in LLC-PK1, and its effect was found to be mediated by attenuation of the phosphorylation of c-Jun N-terminal kinase, MAPK, ERK, p38, and caspase-3 [41]. In particular, we recently found that loliolide, a heterocyclic compound in *M. alba* fruits, showed a protective effect against streptozotocin-induced apoptosis of INS-1 cells via inhibition of the caspase signaling pathway and induction of B-cell lymphoma-2 (Bcl‑2) protein expression, suggesting that the active compound could be effective in preventing diabetes mellitus [42]. In the present study, the ethanol extract of *M. alba* fruits was further investigated to identify potential antidiabetic natural compounds, as there have been few studies on the antidiabetic compounds identified from *M. alba* and their underlying mechanisms of action, although it has been used in traditional medicine to lower blood sugar levels [36], and *M. alba* extracts are known to exhibit antidiabetic properties [37,38]. Phytochemical analysis led to the identification of two main compounds, rutin and quercetin-3-*O*-β-d-glucoside, and their structures were determined by detailed analysis of their nuclear magnetic resonance (NMR) spectroscopic and physical data, which were compared with previously reported values and mass spectrometry (MS) data from liquid chromatography (LC)–MS analysis. Herein, we report the exploration of the antidiabetic and anti-obesity potential of rutin and quercetin-3-*O*-β-d-glucoside (Q3G), focusing on glucose uptake through Akt and AMPK signaling, and lipid accumulation in 3T3-L1 adipocytes.

## 2. Materials and Methods

### 2.1. Plant Material, Extraction, and Isolation

The fruits of *M. alba* were purchased from Woori Herb in Kyungdong Market, the biggest medicinal herb market in Seoul, Korea, in January 2018. The material was authenticated by one of the authors (K.H.K.) and a voucher specimen (SSJ 2018-01) was deposited at the herbarium of the School of Pharmacy, Sungkyunkwan University, Suwon, Korea. The fruits of *M. alba* (5.0 kg) were dried, pulverized, subjected to extraction with 70% aqueous ethanol three times at room temperature, and filtered. The resultant filtrate was evaporated in vacuo to obtain the concentrated residue (720 g), which was dissolved in deionized water and solvent-partitioned with hexane, dichloromethane (CH_2_Cl_2_), ethyl acetate (EtOAc), and *n*-butanol (BuOH, 800 mL, three times). Three solvent-partitioned fractions with increasing polarity were obtained: hexane (13.5 g)-, chloroform (CHCl_3_, 41.2 g)-, and *n*-BuOH (68.3 g)-soluble.

The *n*-BuOH-soluble fraction (65.0 g), the major fraction, was separated using an HP-20 dianion column and washed with water (H_2_O) and methanol (MeOH) to obtain two fractions: MeOH-soluble (8.2 g) and water-soluble (56.8 g) fractions. The MeOH fraction (8.2 g) was separated into five fractions (A–E) using silica gel open-column chromatography with CH_2_Cl_2_:MeOH:H_2_O (9:3:0.1). Fraction B3 (158 mg) was separated using preparative reversed-phase (RP) HPLC equipped with a Waters 996 photodiode array detector using an Agilent Eclipse C18 column (21.2 × 250 mm; flow rate: 5 mL/min) with 30% MeOH to obtain seven subfractions (B31–B37). Fraction B35 (15.5 mg) was purified using semi-preparative Shimadzu prominence HPLC system with SPD-20A/20AV series prominence HPLC UV–Vis detectors using a Phenomenex Luna HPLC phenyl-hexyl column (250 × 10 mm; flow rate: 2 mL/min) with 30% MeOH to isolate Q3G (2.7 mg, *t*_R_ = 34.0 min). Fraction D (0.3 g) was subjected to RP-C_18_ silica gel open-column chromatography with 50% MeOH to obtain four subfractions (D1–D4), and subfraction D4 (43.3 mg) was further separated using the semi-preparative RP HPLC with the Phenomenex Luna HPLC phenyl-hexyl column (250 × 10 mm; flow rate: 2 mL/min) and 18% acetonitrile (MeCN) to yield rutin (3.8 mg, *t*_R_ = 21.5 min).

### 2.2. Chemical Reagents

Dulbecco’s modified Eagle’s medium/high glucose (DMEM/HG), fetal bovine serum (FBS), penicillin–streptomycin, and newborn calf serum were purchased from Thermo Fisher Scientific (Waltham, MA, USA), while 3-(4,5 Dimethylthiazol-2-yl)-2,5 diphenyltetrazolium bromide (MTT), isopropanol, dimethyl sulfoxide (DMSO), and 3-isobutyl-1-methylxanthine (IBMX) were obtained from Merck Millipore (Burlington, MA, USA). Dexamethasone, 10% formalin, phosphate-buffered saline (PBS), insulin, and Oil-Red-O were purchased from Sigma-Aldrich (St. Louis, MO, USA). Rosiglitazone and 2-deoxy-2-((7-nitro-2,1,3-benzoxadiazol-4-yl)amino)-d-glucose (2-NBDG) were purchased from Cayman Chemical (Ann Arbor, MI, USA). Antibodies against β-actin, Akt, phospho-Akt (Ser473), AMPKα, and phospho-AMPKα (Thr172) were purchased from Cell Signaling Technology (Danvers, MA, USA). Peroxidase-conjugated goat anti-rabbit and goat anti-mouse IgG were obtained from Merck Millipore.

### 2.3. 3T3-L1 Preadipocyte Differentiation

To induce differentiation, preadipocytes were allowed to reach confluency (defined as day 0) and then, 2 days later, they were cultured in differentiation initiation medium (DIM) comprising DMEM/HG supplemented with 10 μg/mL insulin, 1 μM dexamethasone, 0.5 mM 3-isobutyl-1-methylxanthine (IBMX), and 10% FBS. After 2 days (defined as day 2), the culture medium was changed to differentiation progression medium (DPM) comprising DMEM/HG possessing 10% FBS and 10 μg/mL insulin for 2 days. Then, on day 4, the cells were maintained in post-differentiation medium (FM) comprising DMEM/HG supplemented with 10% FBS and 1% P/S, which was replaced every 2 days. Adipocytes were allowed to mature for 8 days after initiating differentiation.

### 2.4. Cell Viability Assay

First, 3T3-L1 ells were seeded at a density of 1 × 10^4^ cells/well in 96-well plates for 24 h with complete cellular attachment. Compounds were diluted with medium to the indicated concentrations (5 μM and 10 μM) and added to individual wells with three replicates, followed by 48 h incubation at 37 °C in a humidified atmosphere of 5% CO_2_. Then, 20 μL MTT (5 mg/mL) was added to the sample medium, followed by 2 h incubation. The liquid in the plate was discarded, 100 μL DMSO was added to dissolve the MTT–formazan complex formed by shaking for 10 min, and then the optical density was measured at a wavelength of 540 nm. The percentage viable cells were calculated relative to the viability of untreated, which was set to 100% [43,44].

### 2.5. Glucose Uptake Assay

Glucose uptake into adipocytes was measured using a modified version of the method described by Alonso-Castro et al. [45]. Briefly, on differentiation day 8, adipocytes were incubated for 24 h with the respective test solution in DMEM (serum and glucose-free). After 1 h stimulation with or without insulin, 80 μM of the fluorescent glucose analog 2-NBDG was added, and the culture was incubated for a further 1 h. The uptake of 2-NBDG was stopped by washing the cells with pre-cooled 1 × PBS. The remaining fluorescence activity of the cell monolayers was recorded on a fluorescence microplate reader at wavelengths of 485 (excitation) and 535 (emission) nm. Fluorescence activity in the absence of 2-NBDG was subtracted from all values obtained.

### 2.6. Western Blot Analysis

The cells were washed with ice-cold PBS, collected by centrifugation, and the resulting cell pellet was resuspended in lysis buffer (1× radioimmunoprecipitation assay (RIPA) lysis buffer), 50 mM Tris hydrochloride (HCl), 150 mM sodium chloride (NaCl), 1.0% (*v/v*) 0.1% (*w/v*) sodium dodecyl sulfate (SDS), 1.0 mM ethylenediaminetetraacetic acid (EDTA), NP-40, 0.5% (*w/v*) sodium deoxycholate, 0.01% (*w/v*) sodium azide at a pH 7.4, and 1× protease inhibitor cocktail), followed by incubation on ice for 1 h. After the cell waste was eliminated by centrifugation, the lysate protein concentrations were measured using the Bio-Rad protein assay reagent (Hercules, CA, USA). Cell lysates were then separated using electrophoresis with 10% polyacrylamide gels containing SDS and transferred onto an Immune-Blot polyvinylidene fluoride (PVDF) membrane. The membranes were blocked by incubation in Tris-buffered saline plus 0.01% Tween 20 (TBST) containing 5% nonfat dry milk for 1 h at room temperature. The immunoblots were then incubated with primary antibodies (Akt, AMPK, phospho-Akt, phospho-AMPK, and β-actin; 1:1000 or 1:2000) overnight at 4 °C, followed by secondary antibody (peroxidase-conjugated goat anti-rabbit or goat anti-mouse IgG, 1:2000). The immunoblots were visualized by immersion in an enhanced chemiluminescence (ECL) solution for 5 min, followed by scanning with a ChemiDoc imaging system (Bio-Rad, Hercules, CA, USA).

### 2.7. Oil-Red-O Staining

Lipid accumulation was measured in 3T3-L1 adipocytes using Oil-Red-O staining. Adipocyte differentiation was induced for 8 days, as described above. On day 8, the adipocytes were washed two times with PBS and fixed with 10% formalin for 30 min in a culture plate with wells. Each well was washed with distilled water and stained with a 0.5% Oil-Red-O solution in 100% isopropanol. After 30 min, the cells were washed two times with distilled water and examined under a phase-contrast microscope. Cells were considered lipid-positive when the droplets were stained red. To quantify the cellular lipid content, the Oil-Red-O staining was washed with 100% isopropanol, shaken at room temperature for 30 min, and then the Oil-Red-O in the supernatant was recorded on a SpectraMax^®^ M3 microplate reader at a wavelength of 520 nm (Molecular Devices, San Jose, CA, USA).

### 2.8. Statistical Analysis

All experiments were conducted in triplicate and the data are stated as the means ± standard error of the mean (SEM). Differences between mean values were analyzed by applying the Student’s *t*-test. Statistical significance was set at *p* < 0.05.

## 3. Results

### 3.1. Isolation of Compounds

**Figure 1:**
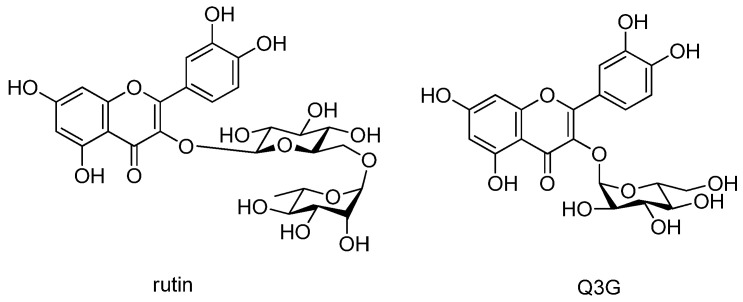
Chemical structures of rutin and quercetin-3-*O*-β-d-glucoside (Q3G)

The *M. alba* fruits were subjected to extraction with 70% aqueous ethanol using a rotary evaporator to obtain the ethanolic extract. The obtained crude extract was sequentially solvent-partitioned using the following four organic solvents with increasing polarity, hexane, CH_2_Cl_2_, EtOAc, and *n*-BuOH, to obtain solvent fractions. LC/MS/UV-based analysis of the fractions revealed that the *n*-BuOH-soluble fraction contains two major peaks with molecular ion peaks at *m*/*z* 611 [M + H]^+^ and *m*/*z* 465 [M + H]^+^ exhibiting a characteristic UV spectrum of flavonoid (λ_max_ 205, 255, and 355 nm). LC/MS/UV-guided fractionation of the BuOH-soluble fraction was performed using repeated open-column chromatography and HPLC purification by LC/MS monitoring to isolate the major peaks. The final semi-preparative HPLC separation monitored by LC/MS yielded two main compounds: rutin [46] and quercetin-3-*O*-β-d-glucoside (Q3G) [47]. Their structures (Figure 1) were determined by comparing their ^1^H and ^13^C NMR spectra with those previously reported in the literature [46,47] and our in-house UV library. NMR spectra were recorded using a Bruker AVANCE III 700 NMR spectrometer operating at 700 MHz (^1^H) and 175 MHz (^13^C). An Agilent 1200 series HPLC system equipped with a diode array detector and a 6130 series electrospray ionization (ESI) mass spectrometer was used for LC/MS analysis, using an analytical Kinetex C_18_ 100 Å column (100 × 2.1 mm, 5 μm; flow rate: 0.3 mL/min). The molecular ions of the compounds detected using LC/MS led us to confirm the structural elucidation. Rutin and Q3G are commercially available. However, the application of LC/MS/UV-guided fractionation used in this study will be helpful to obtain these compounds in significant quantities without the costs.

### 3.2. Effect of Rutin and Q3G on 3T3-L1 Cell Viability

**Figure 2:**
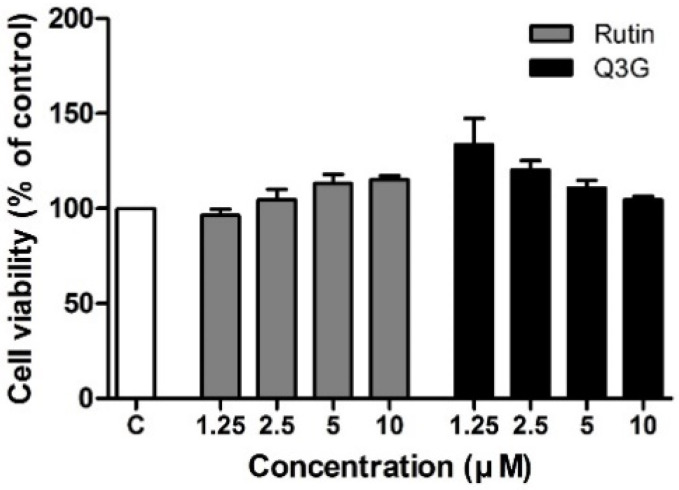
Effect of rutin and quercetin-3-*O*-β-d-glucoside (Q3G) on cell viability of 3T3-L1 adipocytes treated for 48 h. Data are means ± standard error of the mean (SEM, *n* = 3).

The MTT method was used to determine the cell viability of rutin and Q3G in 3T3-L1 preadipocytes cultured for 24 h, followed by incubation with the compounds at a concentration of up to 10 μM for 48 h. Within the range of tested concentrations (1.25–10 µM), rutin- and Q3G-treated cells exhibited similar viability to the untreated control cells (Figure 2). Based on these results, we further studied the two highest concentrations (5 and 10 µM) of each compound.

### 3.3. Effect of Rutin and Q3G on Glucose Uptake in Differentiated 3T3-L1 Adipocytes

**Figure 3:**
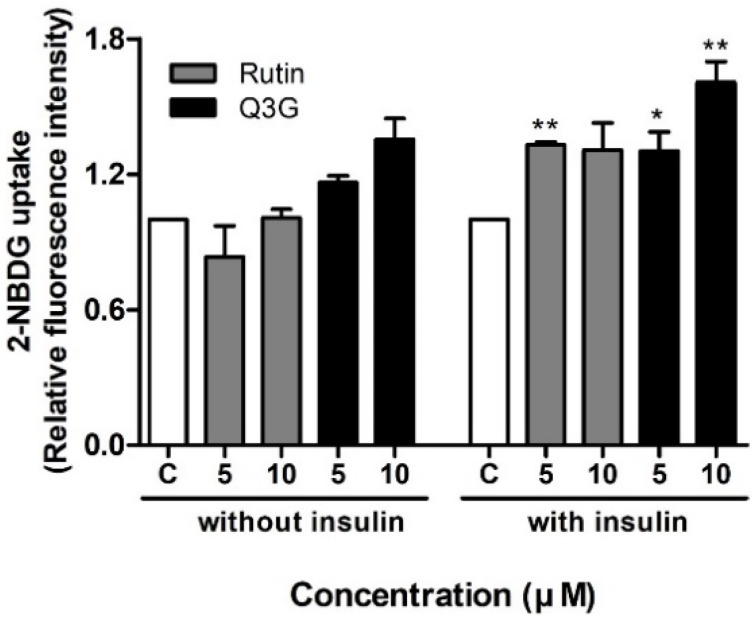
Effect of rutin and quercetin-3-O-β-d-glucoside (Q3G) on basal and insulin-stimulated (100 nM) 2-deoxy-2-((7-nitro-2,1,3-benzoxadiazol-4-yl)amino)-d-glucose (2-NBDG) uptake in differentiated 3T3-L1 adipocytes. Data are means ± standard error of the mean (SEM, *n* = 3); * *p* < 0.05 and ** *p* < 0.01 versus control. C, control.

Glucose transport into adipocytes and other cells is one of the primary metabolic effects mediated by insulin [48]. We investigated the effects of rutin and Q3G on glucose uptake in differentiated 3T3-L1 adipocytes, with or without insulin. As shown in Figure 3, rutin improved insulin-stimulated uptake of 2-deoxy-2-((7-nitro-2,1,3-benzoxadiazol-4-yl)amino)-d-glucose (2-NBDG) by approximately 1.3 times compared to the control group, which only showed statistical significance at 5 μM (*p* < 0.01). Furthermore, Q3G at concentrations of 5 and 10 μM significantly improved glucose uptake by 1.3 (*p* < 0.05) and 1.6 (*p* < 0.01) times, respectively. Relatively higher glucose uptake was also observed with Q3G treatment than with rutin in the absence of insulin, but these differences were not statistically significant.

### 3.4. Effect of Rutin and Q3G on Akt or AMPK Phosphorylation

**Figure 4:**
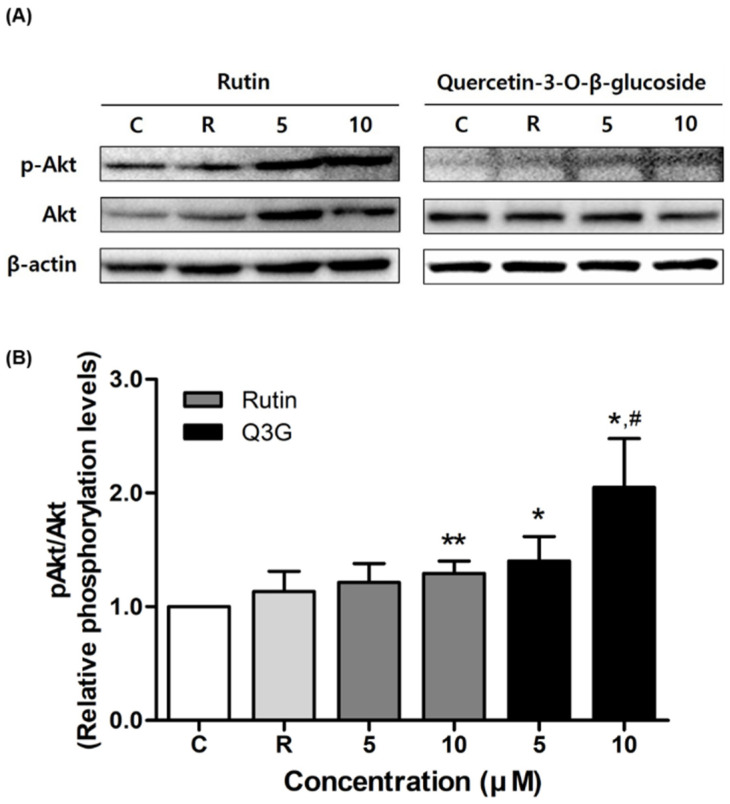
Effect of rutin and quercetin-3-*O*-β-d-glucoside (Q3G) on Akt phosphorylation in insulin-stimulated 3T3-L1 adipocytes. (**A**) Representative Western blots of Akt and phosphorylated (phospho-Akt). (**B**) Quantification of relative phosphorylation levels of Akt. Levels of phospho-Akt were normalized to total Akt for each compound. Data are means ± standard error of the mean (SEM, *n* = 3); * *p* < 0.05 and ** *p* < 0.01 versus control. # *p* < 0.05 versus rosiglitazone 10 μM. C, control; R, rosiglitazone 10 μM.

**Figure 5:**
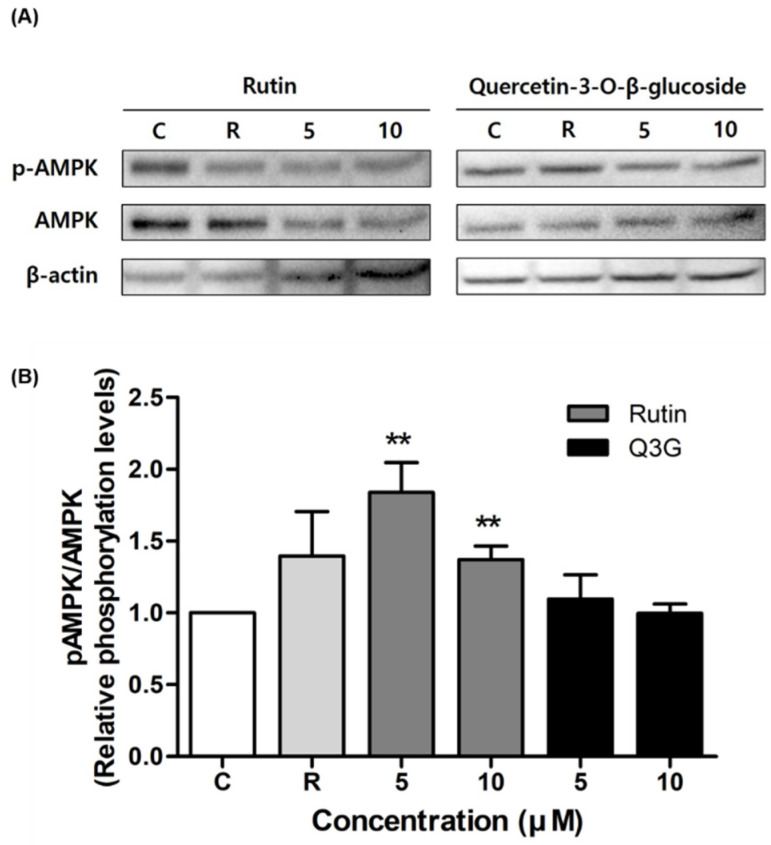
Effect of rutin and quercetin-3-*O*-β-d-glucoside (Q3G) on AMP-activated protein kinase (AMPK) phosphorylation in insulin-stimulated 3T3-L1 adipocytes. (**A**) Representative Western blots for AMPK and phospho-AMPK. (**B**) Quantification of relative phosphorylation levels of AMPK. Phospho-AMPK level was normalized to total AMPK for each compound. Data are means ± standard error of the mean (SEM, *n* = 3); ** *p* < 0.01 versus control. C, control. R, rosiglitazone 10 μM.

To elucidate the mechanism by which rutin and Q3G induce insulin-stimulated glucose uptake, we assessed the activation of Akt and AMPK phosphorylation. As shown in Figure 4, both compounds significantly increased phospho-Akt expression in 3T3-L1 adipocytes. In particular, the Q3G-activated Akt phosphorylation level was 140% (at 5 μM) and 200% (at 10 μM) that of the control level, respectively (both *p* < 0.05). In the case of AMPK phosphorylation, at both concentrations, rutin augmented the expression level of phospho-AMPK in insulin-stimulated 3T3-L1 adipocytes, whereas no significant change was observed with Q3G (Figure 5).

### 3.5. Effect of Rutin and Q3G on Lipid Accumulation

**Figure 6:**
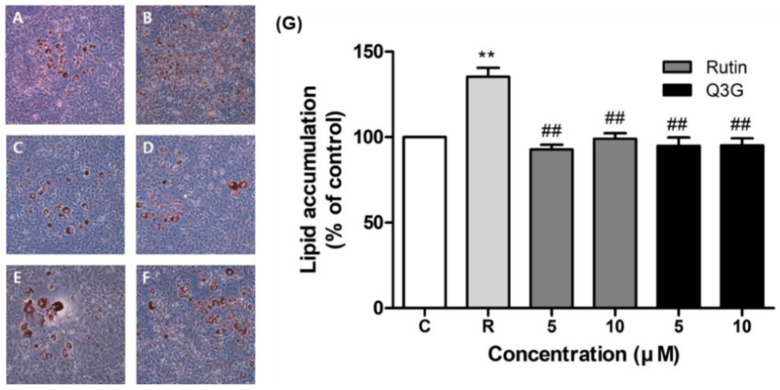
Representative images (magnification ×100) of Oil-Red-O staining in 3T3-L1 cells treated with (**A**) vehicle (control), (**B**) rosiglitazone 10 μM, (**C**) rutin 5 μM, (**D**) rutin 10 μM, (**E**) quercetin-3-*O*-β-d-glucoside (Q3G) 5 μM, and (**F**) Q3G 10 μM (**F**). (**G**) Effect of rutin and Q3G on lipid accumulation in 3T3-L1 adipocytes. Data are means ± standard error of the mean (SEM, *n* = 3); ** *p* < 0.01 versus control and ## *p* < 0.01 versus rosiglitazone 10 μM. C, control; R, rosiglitazone 10 μM.

The effects of rutin and Q3G on lipid accumulation in differentiated 3T3-L1 adipocytes were determined using Oil-Red-O staining (Figure 6). The number of lipid droplets in 3T3-L1 cells increased considerably after treatment with rosiglitazone (Figure 6B), with significantly higher lipid accumulation than in the control (Figure 6G, *p* < 0.01). However, rutin-treated (Figure 6C,D) and Q3G-treated (Figure 6E,F) cells exhibited similar levels of lipid accumulation to the control, indicating that they had no effect on lipid accumulation in 3T3-L1 adipocytes.

## 4. Discussion

Insulin resistance plays a key role in the development of T2D and metabolic syndrome [49], which is a complex and chronic progressive syndrome that leads to adverse effects in many organs. Over the past few years, several studies have revealed that controlling whole-body glucose homeostasis in both normal and diseased states plays an essential role in adipose tissue [50]. Because currently marketed synthetic antidiabetic and anti-obesity agents often cause unexpected adverse effects [51], natural products with fewer side effects are attracting great attention for the treatment of diabetes and obesity. In this study, we investigated whether rutin and Q3G, the major active compounds isolated from *M. alba* fruits, can be used as natural-based therapeutic agents for T2D using a model of 3T3-L1 adipocytes. These two constituents are the major glycosides of quercetin and widely distributed in nature [52]. There have been various studies to determine their health-promoting effects [52], where it has been found that rutin possesses antioxidant, anti-inflammatory, anti-angiogenic, and organ-protective activities [53,54,55,56,57,58,59,60]. Furthermore, a recent review demonstrated that rutin exerts an anti-hyperglycemic effect through the following mechanisms [61]: (1) reduces glucose absorption from the small intestine by α-glucosidases and α-amylase inhibition; (2) increases glucose uptake in muscle and adipose tissue by stimulating GLUT4 synthesis and translocation, activating PI3K- and MAPK-mediated signaling pathways, and increasing peroxisome proliferator-activated receptor gamma (PPARγ) expression; (3) exhibits the reversal of gluconeogenesis by inhibiting the gene expression of key gluconeogenic enzymes, such as glucose-6-phophatase (G6Pase), glycogen phosphorylase, and fructose-1,6-bisphosphatase, and (4) shows suppression of glucotoxicity by IRS2 and AMPK signaling pathway activation. Although relatively few studies on Q3G have been reported, Q3G also exhibited a broad range of physiological properties, including antioxidant, anti-inflammatory, antidiabetic, anti-angiogenic, and chemopreventive activity [52].

Impaired glucose transport is associated with insulin resistance in T2D patients. In this study, the effects of rutin and Q3G on basal and insulin-stimulated glucose uptake into fully differentiated 3T3-L1 adipocytes were examined using non-radioactive measurement of 2-NBDG uptake. The results showed that rutin and Q3G significantly increased insulin-stimulated 2-NBDG glucose uptake. As mentioned above, each glucose transporter isoform controls glucose uptake in a different way. In adipocytes, GLUT4, a representative insulin-sensitive glucose transporter, is translocated from the intracellular vesicle to the cell membrane and modulates glucose utilization [62]. A recent study has reported that the regulation of GLUT4 expression and translocation is affected by various factors, such as adiponectin and insulin signaling mediators [63]. Although we have found that the GLUT4 protein expression in insulin-stimulated 3T3-L1 adipocytes was unchanged after rutin and Q3G treatment (data not shown), these results were limited to total protein expression and further research on the extent of GLUT4 translocation from cytosol to plasma membrane is needed. Insulin signaling upstream proteins, such as insulin receptors, PI3K, should also be evaluated to clarify the mechanisms of the insulin-mimetic actions.

The major mechanism of glucose uptake is the rapid translocation of GLUT4 protein from the cytosol to the plasma membrane through the insulin signaling pathway. In this pathway, the intracellular tyrosine residues of the insulin receptor β-subunit are auto-phosphorylated and activate downstream signaling molecules including IRS-1 and PI3K. Subsequent phosphorylation of Akt promotes the translocation of glucose transporters to the plasma membrane [3,64]. In addition to the insulin signaling pathway, the AMPK-mediated pathway is also involved in glucose transport in muscles and adipocytes [65]. Various AMPK activators, such as 5-aminoimidazole-4-carboxy-amide-1-d-ribofuranoside (AICAR) and metformin, have been reported to enhance glucose uptake in adipocytes through AMPK activation [19,66]. Although these AMPK agonists elevate both insulin-independent and insulin-dependent glucose uptake [67,68], cooperation between AMPK and the insulin signaling pathway on glucose uptake in adipocytes is controversial [69]. In this study, we found that Q3G showed a dose-proportional, specific increase in Akt phosphorylation. To our best knowledge, this is the first report to confirm that Q3G regulates glucose uptake through activation of the Akt-mediated signaling pathway, while rutin significantly increased both Akt and AMPK phosphorylation, which is consistent with a previous study performed in rat pancreatic β cells [70]. This observation suggests the possible simultaneous activation of AMPK and the insulin signaling pathway in controlling glucose uptake.

At the end of adipogenesis, pre-adipocytes (spindle-shaped) change their forms into a round shape, performing various endocrine functions, such as lipid accumulation, glucose uptake promotion in response to insulin, fatty acid synthesis, and secretion of various hormones and cytokines [71]. Thus, intracellular lipid accumulation is generally used as a primary indicator for adipogenesis [72]. The differentiation and maturation of adipocytes and lipid accumulation are also related to the development of obesity [73]. TZDs, such as rosiglitazone, promote insulin-sensitizing action through the activation of PPARγ, which regulates adipocyte differentiation and adipocyte-specific gene expression [74]. Therefore, the use of TZDs for the treatment of T2D can cause unexpected side effects (weight gain) and increase the risk of obesity [75]. Unlike rosiglitazone, rutin and Q3G showed no change in lipid accumulation in 3T3-L1 adipocytes in this study, suggesting that they could be safely used without the risk of body-weight-related side effects and obesity.

## 5. Conclusions

In this study, we isolated rutin and Q3G, as the major active compounds of *M. alba* fruits, and found that these compounds improved glucose uptake via the Akt-mediated insulin signaling pathway and/or AMPK activation. The active compounds also positively affected lipid accumulation in adipocytes, suggesting that glucose uptake was mediated through activation of the Akt and AMPK signaling pathway without induction of adipogenesis. Based on these findings, we conclude that rutin and Q3G, active components of *M. alba* fruits, have the potential to induce fewer side effects such as weight gain than rosiglitazone, an available antidiabetic drug. Therefore, these compounds could be potential therapeutic candidates for the management of T2D.

## Data Availability

Not applicable.
